# A multimodal couple-coping intervention for enhancing sexual adjustment among breast cancer women: Study protocol for a randomised controlled trial

**DOI:** 10.1371/journal.pone.0309218

**Published:** 2024-08-22

**Authors:** Ka Ming Chow, Carmen Wing Han Chan, Kai Chow Choi, Alexandra Leigh McCarthy

**Affiliations:** 1 The Nethersole School of Nursing, Faculty of Medicine, The Chinese University of Hong Kong, Hong Kong, SAR, China; 2 Faculty of Health, Griffith University, Queensland, Australia; UN Mehta Institute of Cardiology and Research Center, INDIA

## Abstract

**Aim:**

To investigate the effects of a multimodal couple-based sexual health intervention for premenopausal women treated for breast cancer and their partners to provide personalised psychosexual care, and to understand participants’ experience of, and adherence to, the intervention.

**Methods:**

This is an assessor-blinded, randomised controlled trial. Premenopausal women treated for breast cancer (N = 160) and their partners will be recruited. Dyads will be randomised into an intervention (n = 80) or attention control (n = 80) group. The intervention group will receive the multimodal couple-based sexual health intervention over eight weeks. The intervention comprises five face-to-face and virtual individual couple counselling sessions combined with online reading, a chat-based discussion forum, and telephone calls. The intervention is based on level I-II evidence and a robust theoretical framework. The attention control group will receive usual care plus telephone calls comprising general greetings and reminders to complete follow-up surveys. Sexual adjustment, relationship quality and quality of life will be measured at baseline, after completion of the intervention, three months and six months post-intervention. The project will adhere to the CONSORT-EHEALTH checklist. Qualitative interviews will explore the participants’ experience of, and adherence to, the intervention.

**Discussion:**

This study will provide the first pragmatic evidence of the effectiveness of a multimodal couple-coping intervention to support premenopausal women and their partners to improve sexuality, relationship quality and quality of life after treatment for breast cancer.

**Implications for the profession and /or patient care:**

Sexual health is a neglected area in clinical practice, for patients and partners. The ever-growing population of women treated for breast cancer at younger age has created a more pressing need for the development of tailored sexual health interventions. If effective, this intervention could be incorporated into routine cancer care to provide better support and care for this patient population to enhance sexual health, intimacy and overall well-being.

**Trial registration:**

ISRCTN35481498; prospectively registered on 08/05/2023.

## 1. Introduction

Breast cancer (BRC) is one of the most prevalent types of cancer, accounting for 24.5% of all cases in women worldwide [1]. BRC refers to cancer originating in breast tissue. BRC can occur in both genders, but 99% occurs in women [2]. Given that the breast is regarded as a symbol of femininity and sexuality in women, a diagnosis of BRC and its related treatment have significant impacts on body image and sexual health [3, 4].

The World Health Organisation (WHO) defines sexual health as physical, emotional, mental and social well-being with respect to sexuality [5]. It has three main dimensions: sexual function, sexual self-concept and sexual relationships [6]. Sexual function embraces the sexual response cycle during intercourse, including sexual desire, excitement, plateau, orgasm and resolution [7]. Sexual self-concept is the image of oneself as a man or a woman, and the evaluation of one’s adequacy in masculine or feminine roles. Sexual relationships are interpersonal situations in which one’s sexuality is shared with another [6]. Sexual health is also intimately connected to body image, which involves thoughts, feelings and behaviours related to one’s appearance and functioning. Body image disturbance resulting from cancer and its treatment includes important sexual aspects of altered appearance, sensory changes and functional impairment [8]. BRC and its treatments, including surgery, chemotherapy, radiation therapy, and endocrine therapy, can have significant adverse effects on sexual health, including body image disruption, reduced sexual desire and arousal, and physical discomfort, ultimately impacting intimacy and relationship quality [4, 9–13].

Women with BRC under age 50 report more treatment-related side effects and are more vulnerable to long-term sequelae [4, 14]. Of BRC cases in Hong Kong, around 27% are diagnosed under age 50, which is the median age women reach menopause [15, 16]. After completion of BRC treatment, only 22% of premenopausal women experience a natural menopause, while the remainder undergoing medication-induced menopause report many sexual problems, including lack of sexual desire, vaginal dryness, and difficulty with arousal, enjoyment or orgasm. These problems often persist beyond five years post-treatment [14]. A study that evaluated the sexual and psychosocial functioning of Chinese women with BRC in Hong Kong found that 59% of the sample (N = 200) were not satisfied with their sexual function after treatment and that a change in partner relations strongly predicted sexual problems that detrimentally affected quality of life (QoL) (OR = 5.25) [17]. Therefore, living with BRC and its aftermath requires considerable sexual adjustment to re-establish and maintain a satisfying sexual relationship so as to improve QoL.

Partners of cancer patients also report many challenges after diagnosis and treatment. One study recruited 122 male and female partners to explore the impact of cancer and its treatment on their sexuality and sexual relationships [18]. The majority of partners (78%) reported changes in sexuality after treatment, such as a complete halt or reduced frequency of sex. Perceptions of loss of intimacy made them feel sad. However, only 20% of the participants had the opportunity to discuss their sexual concerns with healthcare professionals [18]. Intimacy is a key component of relationship quality and could buffer psychological distress [22]. Partners experience psychological distress, but their sexual health needs are neglected [18, 19]. Partners play a crucial role in helping women adjust to their illness, but the caregiving role can lead to relationship strain [11, 18, 20]. Renegotiating sexual health with a supportive partner is necessary to facilitate adjustment to illness [11, 18, 19], rebuild intimacy, and improve QoL [21].

Couple-based interventions refer to interventions delivered to couples currently married or living together [22]. These interventions have yielded significant benefits for sexual health outcomes in other fields and warrant investigation in the BRC context [9, 20, 22]. Evidence from research into the effects of couple-based interventions for sexual problems following BRC has demonstrated positive results on improving sexual function in terms of orgasm and intimacy, sexual and relationship satisfaction, body image, couple communication, dyadic coping, adjustment and QoL of both patients and their partners [9, 20, 22]. Providing and receiving support from each other during couple-based interventions promotes joint problem-solving and shared coping [20]. Positive partner coping enhances sexual adjustment and achieves a positive couple relationship [22]. Conventionally, couple-based interventions to date have been delivered in face-to-face mode. However, with the popularity and increased recognition of electronic usage of healthcare services, online-based or mobile interventions are potentially viable alternatives to face-to-face delivery of sexual healthcare [23, 24]. It is suggested to be more effective if the delivery mode is adjusted to the needs of patients. In view of the importance of establishing a rapport between clinician and patient, a combination of face-to-face and online modes would be appropriate to address sexual health concerns in a comfortable atmosphere [23]. Yet, there is a paucity of evidence to support the use of tailored mixed mode delivery in addressing sexual health concerns.

The incidence of BRC and its impact on sexual function and QoL have indicated a pressing need for couple-based intervention to address sexual health issues after BRC treatment. However, there are no easily accessible sexual health services tailored to individuals treated for cancer and their partners in Hong Kong. Existing sexual health services are mainly provided by not-for-profit organisations or private clinics targeting common physiological-based sexual and reproductive issues in the general population. The ever-growing population of women treated for BRC at a younger age has created a more pressing need for the development of tailored sexual health interventions that cater for their distinctive needs. As such, it is timely to develop an empirically-based and theory-driven Multimodal Couple-coping Intervention (MCI) to address sexual health concerns among premenopausal women and their partners after BRC treatments.

This study aims to evaluate the effects of the MCI on sexual adjustment among premenopausal women treated for BRC, and relationship quality and quality of life among the women and their partners.

## 2. Materials and methods

### 2.1 Trial design

**Fig 1:**
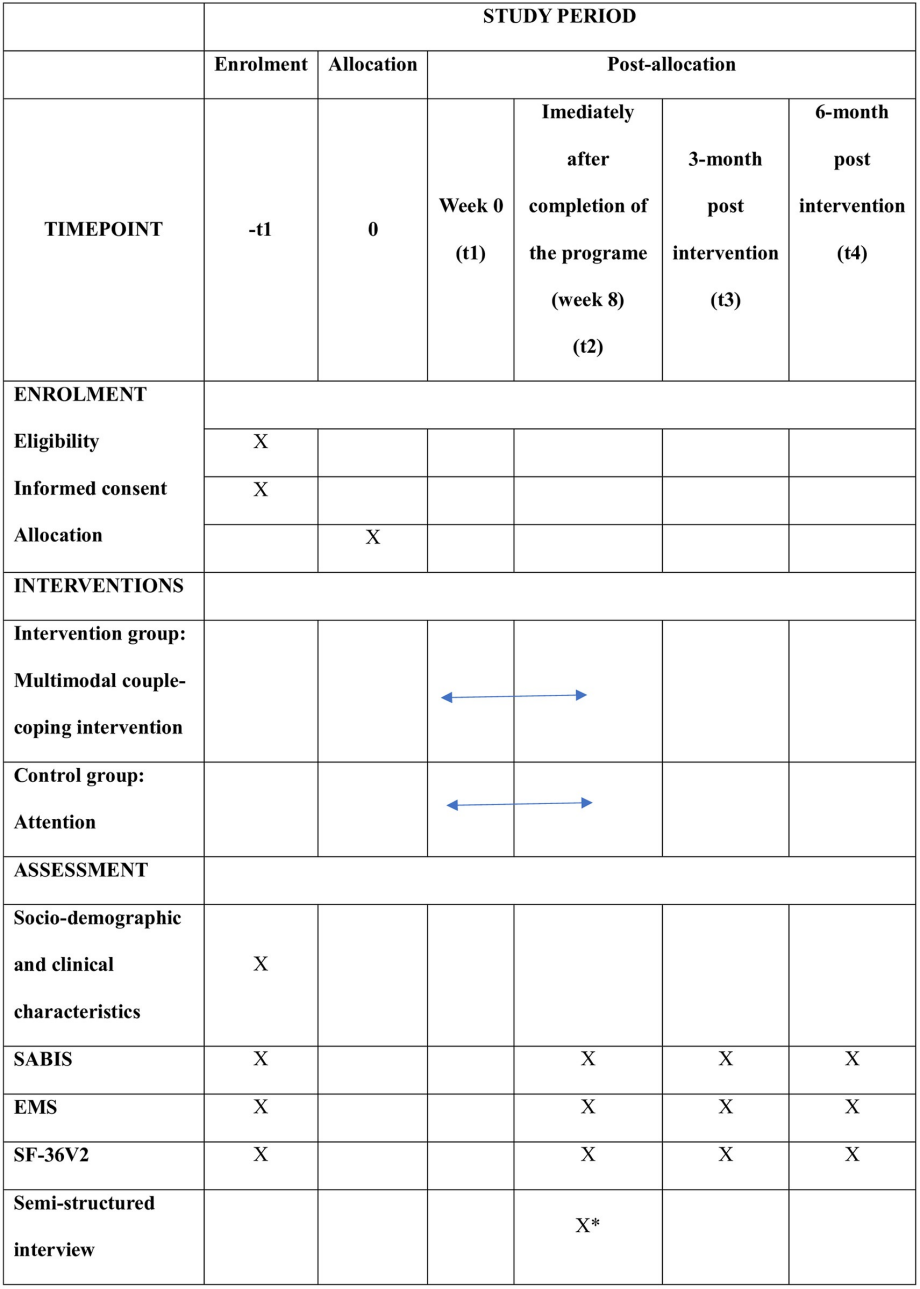
SPIRIT schedule of enrolment, interventions, and assessments. Note: * *Intervention group only*, *SABIS*, *Sexual Adjustment and Body Image Scale; EMS*, *ENRICH Marital Satisfaction Scale; SF-36v2*, *MOS 36-item Short From version 2*.

This an assessor-blind, parallel randomised controlled trial (RCT). This study protocol adheres to the guidelines of the Standard Protocol Items: Recommendations for Intervention Trials (SPIRIT) [25] (Fig 1, S1 Checklist). The full study will adhere to the CONSORT-EHEALTH checklist [26] (S2 Checklist).

### 2.2 Intervention

**Fig 2:**
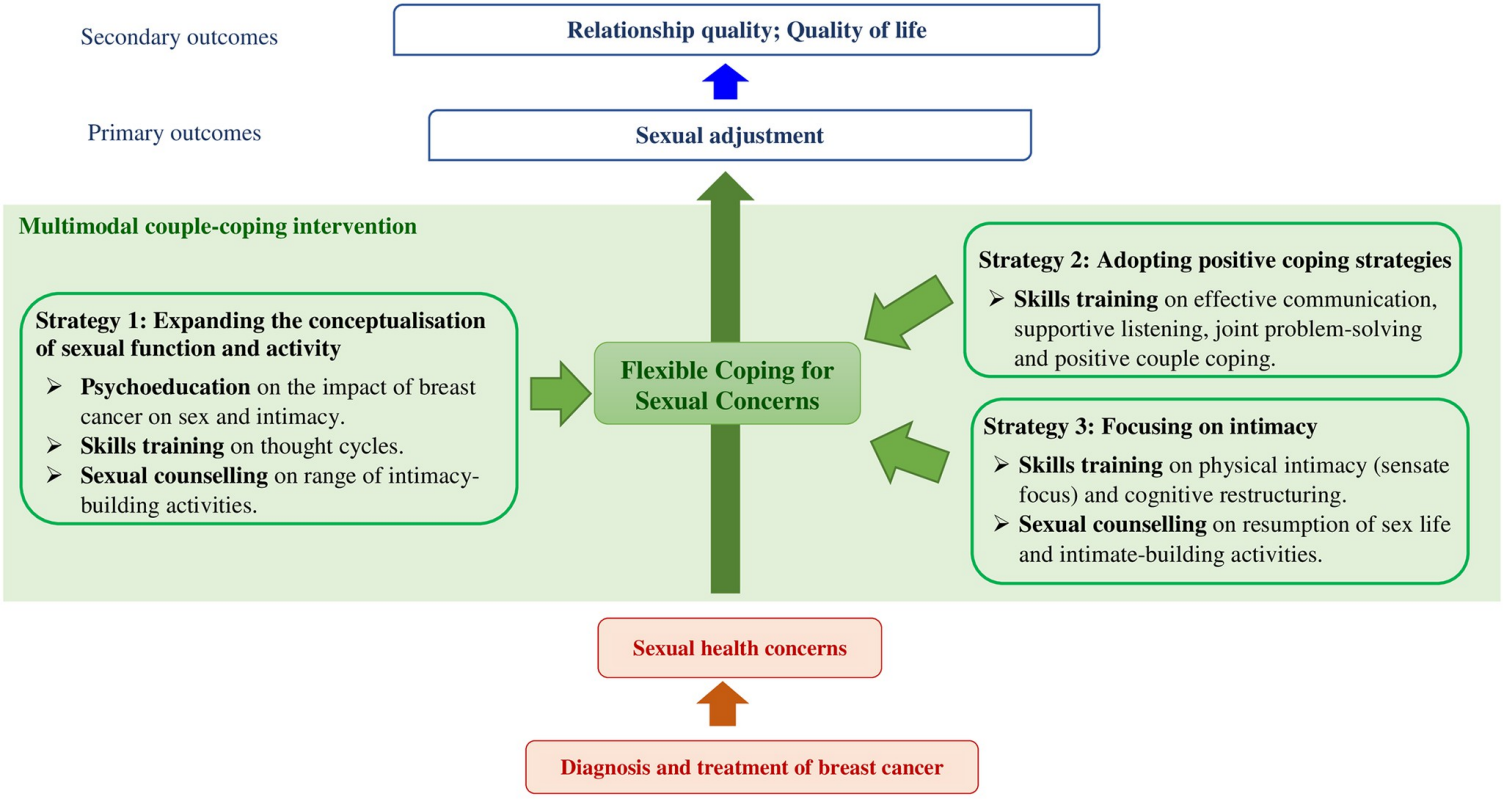
The conceptual framework of the multimodal couple-coping intervention. Note: This conceptual framework is adapted from the Model of Flexible Copying with Sexual Concerns (Reese et al., 2010).

Flexible Coping with Sexual Concerns provides the philosophical underpinning of the proposed MCI. It enables responsive intervention delivery, adapted to need, by expanding the conceptualisation of sexual function and activity, adopting positive coping strategies, and shifting the focus on sexual functioning to intimacy, subsequently bringing beneficial effects on sexual adjustment and relationship satisfaction [27]. It is posited that a discussion of flexibility in coping with sexual health concerns early in the treatment trajectory facilitates sexual adjustment and the renegotiation of intimate relationships during survivorship. The intervention components integrate evidence-based techniques from cognitive behavioural couple therapy and sex therapy for intimacy enhancement [28], combined with psychoeducation, skills training and therapeutic counselling [22], in order to motivate beneficial cognitive and behavioural changes in sexual function and activity [27]. With flexible coping with sexual concerns, individuals can think about sexual activity differently and engage in attainable sexual activities to maintain and foster intimacy [27]. The causal model is illustrated in Fig 2.

According to the model, a more flexible perception of sexuality for individuals who may not be able to resume or engage in their previous level and type of sexual activity is beneficial. In the context of BRC, enhancing the level of flexibility among women treated for BRC and their partners has the potential to promote their sexual adjustment, improve relationship quality and QoL. To increase flexibility, the intervention will adopt three strategies: expanding conceptualisation of sexual function and activity, adopting positive coping strategies, and focusing on intimacy [27]. The components of the MCI include psychoeducation, skills training and therapeutic counselling with the integration of techniques from cognitive behavioural couple therapy and sex therapy [22, 28]. The contents and design of the intervention are also guided by the findings of previous studies [20, 22, 27, 28], and the successful completion of the project led by the authors in which factors that might enhance the provision of sexuality care and the information needs of sexual health and preferences for sexuality care of female gynaecological cancer patients were identified [29]. The information needs include the possibility and suitable timing of sex life resumption, its potential impact on physical health, adaptation to changes in sex life, and potential impact of disease and treatment on physical, psychological and sexual functioning. Regarding the format, the participants indicated the preference for face-to-face counselling with partner participation during post-operation and rehabilitation, and information delivered via the internet with reading materials [29].

The proposed MCI is an eight-week five-session programme, each session delivered every two weeks (at Weeks 0, 2, 4, 6, and 8), covering topics on the impact of BRC on sex and intimacy, communication and problem-solving skills, dyadic positive coping, cognitive restructuring of sex and intimacy, intimacy-building activities, and maintaining intimacy [20, 22, 27–29]. The first and last sessions will be conducted in face-to-face dyad format, the second session will be virtual dyad counselling, the third one will be online reading with a chat-based discussion forum and homework exercise, and the fourth will be a reminder telephone call for reviewing what has been achieved and scheduling the last session. The counselling sessions will last for 60–90 minutes and will be delivered by a full-time research nurse who will receive one-week of training from the first author to provide the intervention, while the online discussion will be moderated by the research nurse to monitor the progress of online reading. The telephone call will last for around 30 minutes. The components and contents of the MCI are listed in Fig 2 and Table 1.

**Table 1:** Design of the multimodal couple-coping intervention.

Session (format)	Components	Topic and content	Strategies
1 (in-person dyad counselling)	Psychoeducation & skills training	*Impact of breast cancer (BRC) on sex and intimacy* Effects of BRC on sex and intimacy Possibility and impact of resumption of sex life Couple relationship history Sexual response cycle Sexual adjustment and body image Intimacy challenges and goal-setting Introduction to physical intimacy skills (sensate focus)	1,3
2 (virtual dyad counselling)	Skills training & therapeutic counselling	*Enhancing intimacy through communication* Effective communication about sex and intimacy (problem-solving and feelings expression) Positive coping skills Challenges in communicating effectively about sex and intimacy Communication skills practice	2,3
3 (online reading materials & chat-based discussion)	Skills training & therapeutic counselling	*Enhancing intimacy through cognitive restructuring* Positive and negative thought cycles Flexible and inflexible thinking about sex and intimacy Identifying and changing negative/ inflexible thoughts Broadening range of intimate activities (intimacy-building activities)–sensate focus	1,3
4 (reminder telephone call)	Psychoeducation & therapeutic counselling	*Enhancing intimacy through sensate focus* Review of communication skills and sensate focus Evaluate progress of sensate focus practice	1,3
5 (in-person dyad counselling)	Psychoeducation, skills training & therapeutic counselling	*Planning ahead for intimacy* Review of skills and psychoeducation covered Evaluate progress towards goals Plan for continues skills practice Anticipate and plan for challenges (maintaining intimacy) Sexual counselling on resumption of sex life and intimate activities	1,2,3

### 2.3. Study setting and participants

We will recruit women treated for BRC and their partners in the oncology clinics of three regional hospitals in Hong Kong during their regular follow-ups. The inclusion criteria include (1) women with a diagnosis of BRC; (2) all active treatment completed in the previous six months but endocrine and/ or maintenance therapy allowed with no evidence of metastatic disease, so that the intervention will be delivered to them during early survivorship, with the intent to prepare the women and their partners for life after cancer; (3) with a regular sexual partner, either heterosexual or homosexual; (4) over 18 years; (5) in a premenopausal state when diagnosed with BRC; (6) has a smartphone with internet connection; (7) able to understand spoken Cantonese and to read Chinese; (8) consenting to participate. Those with a known pre-existing psychiatric illness were excluded.

The sample size will adequately power the study to evaluate the effects of the MCI on the outcomes of sexual adjustment, relationship quality and QoL. To our knowledge, no study has investigated the effects of a multimodal couple-based intervention on sexual health outcomes. Without an empirically-derived effect size to guide our sample size planning, we instead define a clinically-relevant change as a 0.5 standard deviation (SD) improvement in the above outcomes on an *a priori* basis. Such an effect corresponds to a medium effect size [30] and is empirically regarded as a minimally important difference [31]. By using the power analysis software PASS 16 (NCSS, LLC. Kaysville, Utah, USA), it is estimated based on an independent t-test that a sample size of 64 dyads per group is adequate to achieve 80% power at a two-sided 5% level of significance to detect an effect size of 0.5 SD in an outcome at a post-intervention time point. Further allowing for an overall attrition rate up to 20% [22], a total of 160 eligible dyads, with 80 per group will be recruited into the RCT.

### 2.4 Recruitment

A research nurse will approach eligible women and their partners in the oncology clinic during the period of 1 November 2023 to 31 December 2024 and explain the aims of the study to them. Information sheets about the study and consent forms will be provided. After written consent is obtained, the participating dyad’s demographic data will be collected, and the instruments will be administered during baseline face-to-face interviews before randomisation.

### 2.5 Randomisation and blinding

The participants will be randomly assigned to either the intervention or attention control groups. Subject allocation will be performed using stratified block randomisation with a block size of 10. A random sequence of grouping identifiers (I or C) based on computer-generated random numbers will be prepared in advance by an independent statistician for each oncology clinic. The grouping sequence lists will be password-protected and stored in a computer, and only be accessible to staff independent of the study or responsible for group allocation. The group allocation of each participant will be assigned according to the sequence of enrolment and the corresponding group identifier in the prior prepared random sequence list.

To guarantee that the outcome assessor is blinded, a research assistant who is uninformed about the group status of the participants will be responsible for conducting reassessments. Reassessment will be conducted at the same time point for both groups, i.e., on the completion of the intervention, three months and six months post-intervention via telephone call. Participating dyads in the intervention group will be invited to be interviewed about their experiences and feelings towards the intervention. Another research nurse will perform qualitative interviews with the participating dyads to avoid an experimenter effect.

### 2.6 Control intervention

While the participants in the intervention group will receive the MCI, those in the attention control group will receive attention from the research nurse five times during the same period as the intervention group to prevent bias from the special attention given. The research nurse will only approach the participants to invite them to participate in the study and collect baseline data, but they will not provide any form of intervention to them. The research nurse will contact the participants through phone calls at weeks 2, 4, 6, and 8, where they will deliver general greetings and provide general advice.

### 2.7 Outcome measures

#### 2.7.1 Primary outcome

The Chinese version of the sexual adjustment scale of the Sexual Adjustment and Body Image Scale (SABIS) will be used to assess the effect of the MCI on sexual adjustment. The SABIS consists of two subscales: a six-item body image scale and an eight-item sexual adjustment scale. Using a five-point Likert scale with scores ranging from 1 to 5, higher scores indicate better outcomes. The scale has been used previously in BRC with good reliability and validity [10]. The Chinese SABIS has demonstrated good construct validity, internal consistency, and test-retest reliability among Chinese patients with BRC [32].

#### 2.7.2 Secondary outcomes

The Chinese version of ENRICH Marital Satisfaction Scale (EMS) will be used to assess the quality of partner relationships of the participating dyads individually. It consists of 10 items, extracted from the original full-length 125-item ENRICH Marital Inventory, which are recognised as the most important dimensions of relationships by the original author. A five-point Likert scale with scores ranging from 1 to 5 is used, with higher scores indicating better relationships. The scale has established discriminant validity and demonstrated good reliability, with an internal consistency of 0.76 among Chinese couples [33].

QoL of the participating dyads will be measured by the Hong Kong Chinese version of the MOS 36-item Short Form (SF-36) v2 health survey. The 36 items are clustered into eight domains: physical functioning (PF), role physical (RP), bodily pain (BP), general health (GH), vitality (VT), social functioning (SF), role emotional (RE), and mental health (MH). All items in each domain are summed and transformed into scales from 0 to 100. Furthermore, the PF, RP, BP, and GH domains are aggregated into the physical health component summary (PCS) score, and the VT, SF, RE, and MH domains are aggregated into the mental health component summary (MCS) score. Higher scores indicate better QoL. The Chinese (HK) version of the SF-36v2 has demonstrated good construct validity, internal consistency and test-retest reliability among the general Chinese adult population in Hong Kong [34].

#### 2.7.3 Other measurements

A data collection sheet will record the socio-demographic and clinical characteristics of the BRC women: age, education level, monthly household income, marital status, duration of marriage/current relationships, number of children, religious beliefs, stage of cancer, time since diagnosis, treatment modality and length of treatment. Socio-demographic data of the partners will also be collected: age, education level, medical and drug history.

Qualitative data will be collected from the intervention group through semi-structured interviews up to the point of data saturation, estimated to be 20 participating dyads. Their experiences and perceptions of the MCI, and whether the programme improved their sexual adjustment will be explored. The interviews will be scheduled after the completion of the programme and audiotaped with consent.

### 2.8 Data collection procedure

A research nurse will be responsible for subject recruitment, randomisation, and baseline assessment which uses the socio-demographic and clinical characteristics, the Chinese version of the SABIS, EMS, and SF-36v2. The SABIS, EMS, and SF-36v2 will be reassessed immediately after the completion of the programme, three months and six months post-intervention. To ensure an unbiased assessment, a research assistant who is unaware of the participants’ group assignment will conduct phone interviews for the reevaluation. Another research nurse will be responsible for conducting semi-structured interviews with participants in the intervention group immediately after the final session of the programme (Fig 1).

### 2.9 Data analysis

Data will be summarised and presented using appropriate descriptive statistics. Continuous and categorical data will be presented in mean (standard deviation) and frequency (percentage), respectively. The homogeneity of participants’ characteristics between the two study arms will be assessed using independent t, Mann-Whitney, chi-square or Fisher’s exact tests, as appropriate. Intention-to-treat principle will be adopted in the outcome analysis by using a generalised estimating equations (GEE) model to compare the differential changes in each outcome across the four time points between the two arms with adjustment for the stratifying factor (oncology clinic). The interventional effects on the outcomes are indicated by the group-by-time interaction terms in the GEE analyses. All statistical analyses will be performed using IBM SPSS version 28 (IBM Crop., Armonk, NY). All statistical tests are two-sided with level of significance set at 0.05.

Audiotapes of the interviews will be examined by means of thematic analysis. First, the recorded tapes will be transcribed verbatim. All transcripts will be compared with the recording to check for any discrepancies and revised as necessary. The transcripts will then be content analysed to identify persistent words and code themes within the data. Lastly, significant categories, theoretical themes and linkages within the data will be formulated and confirmed by the first author and a co-author to improve its accuracy, and then translated into English [35].

### 2.10 Validity and reliability

This protocol has been reviewed and approved by the General Research Fund (GRF) of the Research Grants Council of Hong Kong. The effects of this evidence-based and theory-guided intervention will be evaluated by the gold standard research design (RCT), using validated instruments and appropriate statistical analysis. The assessor will be blinded to ensure that outcomes are evaluated objectively and independently. These practices will enhance the validity and reliability of the study findings, reducing potential bias and enhancing generalisation.

### 2.11 Ethical considerations

This protocol has been reviewed and approved by the Research Ethics Committees of the participating hospitals (approval numbers: 2022.416-T; HKECCREC-2022-061). The first author will report annually to the institutional review board about the progress of this study and keep them informed of any revisions to the protocol, if applicable. The International Standard Randomised Controlled Trial (ISRCTN) registration number for this study is ISRCTN35481498. This study will follow the Declaration of Helsinki and ICH-GCP. Participation is completely voluntary, and participants can withdraw from the study at any time without explanation or penalty. Written informed consent from participants will be obtained prior to the collection of data. This research will not cause any harm to participants. Each participant will be allocated with a code and, all their information and data will be kept confidential. The data files will be kept on a password-protected computer, while the paper documents will be kept stored securely in locked cabinets which will be destroyed five years after completion of the study. The electronic data will be kept for 10 years after the completion of the study. Only the researchers and research staff will have access to the data, which will only be used for research purposes. The findings will be disseminated to the appropriate healthcare agencies and a full report will be provided to the service providers. Additionally, the findings will be presented at professional conferences and published in local and international refereed journals.

## 3. Discussion

This is the first RCT of a multimodal couple-coping intervention to provide personalised psychosexual care after treatment for premenopausal BRC in Hong Kong. If the intervention is deemed effective, it will contribute to the existing literature of nursing interventions that address sexual health issues and aim to support premenopausal women with BRC and their partners to improve relationship quality and QoL. This research study has the potential to provide valuable insights and evidence-based support for healthcare professionals working with couples facing these challenges. Additionally, it may pave the way for future interventions and treatments targeting similar populations in the region.

### 3.1 Limitations

This study has potential limitations that need to be acknowledged. Firstly, due to the nature of the study, it is not possible to blind the participants and the intervener as they actively participate in the intervention. This lack of blinding introduces the potential for bias as participants might have knowledge of their intervention group, which can influence the study results. However, in order to minimise bias, the outcome assessors will be blinded to ensure impartial evaluation. Secondly, there is a risk of loss to follow-up during the 3-month and 6-month period after completion of the intervention, which can affect the statistical power of the study. To address this issue, the sample size calculation anticipates attrition of 20%, and intention-to-treat will be adopted during data analysis.

## 4. Conclusion

The findings of this study will make a valuable contribution to the existing theoretical and practice knowledge regarding the effectiveness of a multimodal couple-coping intervention in supporting premenopausal BRC patients and their partners to enhance sexual adjustment, relationship quality, and QoL. If the intervention demonstrates positive results, nurses can incorporate it into routine patient care to address and alleviate concerns related to sexual health. By implementing this intervention, nurses can provide comprehensive support to couples affected by BRC and help improve their overall well-being and relationship dynamics.

## Supporting information

S1 Checklist SPIRIT checklist. (PDF)

S2 Checklist CONSORT-EHEALTH checklist. (PDF)

S1 Protocol Study protocol. (PDF)

## References

[1] Sung H, Ferlay J, Siegel RL, Laversanne M, Soerjomataram I, Jemal A, et al. Global Cancer Statistics 2020: GLOBOCAN estimates of oncidence and mortality worldwide for 36 cancers in 185 countries. CA Cancer J Clin. 2021;71(3):209–49. doi: 10.3322/caac.21660 33538338

[2] World Health Organization. Breast cancer 2023 [cited 2023 Sep 4]. https://www.who.int/news-room/fact-sheets/detail/breast-cancer.

[3] Runowicz CD, Leach CR, Henry NL, Henry KS, Mackey HT, Cowens-Alvarado RL, et al. American Cancer Society/American Society of Clinical Oncology Breast Cancer Survivorship Care Guideline. CA Cancer J Clin. 2016;66(1):43–73. doi: 10.3322/caac.21319 26641959

[4] Jeng CJ, Hou MF, Liu HY, Wang LR, Chen JJ. Construction of an integrated sexual function questionnaire for women with breast cancer. Taiwan J Obstet Gynecol. 2020;59(4):534–40. doi: 10.1016/j.tjog.2020.05.011 32653125

[5] WHO. Sexual health: Definition 2023 [cited 2023 Sep 5]. https://www.who.int/health-topics/sexual-health#tab=tab_2.

[6] Woods NF. Toward a holistic perspective of human sexuality: Alterations in sexual health and nursing diagnoses. 1987;1(4). 10.1097/00004650-198708000-00004 3648053

[7] Chen C-H, Lin Y-C, Chiu L-H, Chu Y-H, Ruan F-F, Liu W-M, et al. Female sexual dysfunction: definition, classification, and debates. Taiwan J Obstet Gynecol. 2013;52(1):3–7. doi: 10.1016/j.tjog.2013.01.002 23548211

[8] Fingeret MC, Teo I, Epner DE. Managing body image difficulties of adult cancer patients: lessons from available research. Cancer. 2014;120(5):633–41. doi: 10.1002/cncr.28469 24895287 PMC4052456

[9] Carroll AJ, Baron SR, Carroll RA. Couple-based treatment for sexual problems following breast cancer: a review and synthesis of the literature. Support Care Cancer. 2016;24(8):3651–9. doi: 10.1007/s00520-016-3218-y 27154014

[10] Dalton EJ, Rasmussen VN, Classen CC, Grumann M, Palesh OG, Zarcone J, et al. Sexual Adjustment and Body Image Scale (SABIS): a new measure for breast cancer patients. Breast J. 2009;15(3):287–90. doi: 10.1111/j.1524-4741.2009.00718.x 19645784 PMC4856437

[11] Mendoza N, Molero F, Criado F, Cornellana MJ, González E. Sexual health after breast cancer: Recommendations from the Spanish Menopause Society, Federación Española de Sociedades de Sexología, Sociedad Española de Médicos de Atención Primaria and Sociedad Española de Oncología Médica. Maturitas. 2017;105:126–31. doi: 10.1016/j.maturitas.2017.02.010 28268037

[12] Bower JE, Ganz PA, Aziz N, Fahey JL. Fatigue and proinflammatory cytokine activity in breast cancer survivors. Psychosom Med. 2002;64(4). doi: 10.1097/00006842-200207000-00010 12140350

[13] Oflazoğlu U, Varol U, Alacacıoğlu A, Aşık N, Salman T, Taşkaynatan H, et al. The effect of adjuvant chemotherapy on sexual satisfaction and quality of life in breast cancer atients and their partners Izmir Oncology Group (IZOG) Study. Acta Oncologica Turcica. 2018;51(3):357–62. doi: 10.5505/aot.2018.29494

[14] Bloom JR, Stewart SL, Chang S, Banks PJ. Then and now: quality of life in young breast cancer survivors. Psychooncology. 2004;13(3):147–60. doi: 10.1002/pon.794 15022150

[15] Hong Kong Cancer Registry-Hospital Authority. Female breast cancer in 2020. 2023 [cited 2023 Sep 4] https://www3.ha.org.hk/cancereg/pdf/factsheet/2020/breast_2020.pdf

[16] Family Health Service—Department of Health—The Government of the Hong Kong Special Administrative Region. When does menopause usually take place? 2013 [cited 2023 Sep 4]. https://www.fhs.gov.hk/english/health_info/faq/women_health/WH2_5_5.html.

[17] Zee B, Huang C, Mak S, Wong J, Chan E, Yeo W. Factors related to sexual health in Chinese women with breast cancer in Hong Kong. Asia Pac J Clin Oncol. 2008;4(4):218–26. doi: 10.1111/j.1743-7563.2008.00214.x

[18] Hawkins Y, Ussher J, Gilbert E, Perz J, Sandoval M, Sundquist K. Changes in sexuality and intimacy after the diagnosis and treatment of cancer: the experience of partners in a sexual relationship with a person with cancer. Cancer Nurs. 2009;32(4). doi: 10.1097/NCC.0b013e31819b5a93 19444088

[19] Gilbert E, Ussher JM, Perz J. Renegotiating sexuality and intimacy in the context of cancer: the experiences of carers. Arch Sex Behav. 2010;39(4):998–1009. doi: 10.1007/s10508-008-9416-z 19067153

[20] Zimmermann T. Intimate relationships affected by breast cancer: interventions for couples. Breast Care (Basel). 2015;10(2):102–8. doi: 10.1159/000381966 26195938 PMC4464013

[21] Moreira H, Canavarro MC. Psychosocial adjustment and marital intimacy among partners of patients with breast cancer: a comparison study with partners of healthy women. Psychosoc Oncol. 2013;31(3):282–304. doi: 10.1080/07347332.2013.778934 23656256

[22] Li Q, Loke AY. A systematic review of spousal couple‐based intervention studies for couples coping with cancer: direction for the development of interventions. Psychooncology. 2014;23(7):731–9. doi: 10.1002/pon.3535 24723336

[23] Kang HS, Kim H-K, Park SM, Kim J-H. Online-based interventions for sexual health among individuals with cancer: a systematic review. BMC Health Serv Res. 2018;18(1):167. doi: 10.1186/s12913-018-2972-6 29514669 PMC5842558

[24] Karim H, Choobineh H, Kheradbin N, Ravandi MH, Naserpor A, Safdari R. Mobile health applications for improving the sexual health outcomes among adults with chronic diseases: a systematic review. Digit Health. 2020;6:2055207620906956. doi: 10.1177/2055207620906956 32128234 PMC7036501

[25] Chan A-W, Tetzlaff JM, Altman DG, Laupacis A, Gøtzsche PC, Krleža-Jerić K, et al. SPIRIT 2013 Statement: defining standard protocol items for clinical trials. Ann Intern Med. 2013;158(3):200–7. doi: 10.7326/0003-4819-158-3-201302050-00583 23295957 PMC5114123

[26] Eysenbach G, CONSORT-EHEALTH Group. CONSORT-EHEALTH: improving and standardizing evaluation reports of Web-based and mobile health interventions. J Med Internet Res. 2011;13(4):e126. doi: 10.2196/jmir.1923 22209829 PMC3278112

[27] Reese JB, Keefe FJ, Somers TJ, Abernethy AP. Coping with sexual concerns after cancer: the use of flexible coping. Support Care Cancer. 2010;18(7):785–800. doi: 10.1007/s00520-010-0819-8 20165890 PMC5679275

[28] Reese JB, Zimmaro LA, Lepore SJ, Sorice KA, Handorf E, Daly MB, et al. Evaluating a couple-based intervention addressing sexual concerns for breast cancer survivors: study protocol for a randomized controlled trial. Trials. 2020;21(1):173. doi: 10.1186/s13063-019-3975-2 32051002 PMC7014745

[29] Chow KM, Chan CWH, Choi KC, White ID, Siu KY, Sin WH. A practice model of sexuality nursing care: a concept mapping approach. Support Care Cancer. 2021;29(3):1663–73. doi: 10.1007/s00520-020-05660-1 32767106

[30] Cohen J. Quantitative methods in psychology: a power primer. Psychol Bull. 1992;112:1155–9. doi: 10.1037//0033-2909.112.1.155 19565683

[31] Norman GR, Sloan JA, Wyrwich KW. Interpretation of changes in health-related quality of life: the remarkable universality of half a standard deviation. Med Care. 2003;(5):582–92. doi: 10.1097/01.MLR.0000062554.74615.4C 12719681

[32] Zhang P, Chen F, Lian X, Ding S, Zhao Z, Lin X. 中文版乳腺癌患者性调节和身体意象量表的评价 [Validation of the Chinese version Sexual Adjustment and Body Image Scale (SABIS) for breast cancer patients]. Journal of Nursing Science. 2013;28:34–6. Chinese.

[33] Shen ACT. 西方婚姻滿意量表對台灣婚姻關係量之適用性[The applicability of Western marital satisfaction measures for couples in Taiwan based on ENRICH]. Psychological Testing. 2021;48(2):131–51. Chinese.

[34] Lam CLK. Reliability and construct validity of the Chinese (Hong Kong) SF-36 patients in primary care. Hong Kong Practitioner. 2003;25(10):468.

[35] Twinn S. An exploratory study examining the influence of translation on the validity and reliability of qualitative data in nursing research. J Adv Nurs. 1997;26(2):418–23. doi: 10.1046/j.1365-2648.1997.1997026418.x 9292378

