# Supplementary material for: A multimodal couple-coping intervention for enhancing sexual adjustment among breast cancer women: Study protocol for a randomised controlled trial
Source: PLoS One. 2024 Aug 22;19(8):e0309218. doi: 10.1371/journal.pone.0309218 (PMC11340947; doi:10.1371/journal.pone.0309218)
Supplement: S1 Protocol — (PDF) [file pone.0309218.s003.pdf]

PROTOCOL

Study title: Randomised controlled trial of a multimodal couple-coping intervention to enhance couples' sexual adjustment after treatment for premenopausal breast cancer

i. Research context

Breast cancer (BRC) refers to cancer originating from breast tissue. BRC can occur in both genders, 99% of BRC occurs in women [1]. Given that the breast is regarded as a symbol of femininity and sexuality in women, a diagnosis of BRC and its related treatment have significant impacts on body image and sexual health [2,3].

The World Health Organisation (WHO) defines sexual health as physical, emotional, mental and social well-being with respect to sexuality [4]. It has three main dimensions: sexual function, sexual self-concept and sexual relationships [5]. Sexual function embraces the sexual response cycle during intercourse, including sexual desire, excitement, plateau, orgasm and resolution [6]. Sexual self-concept is the image of oneself as a man or a woman, and the evaluation of one's adequacy in masculine or feminine roles. Sexual relationships are the interpersonal situations in which one's sexuality is shared with another [5]. Sexual health is also intimately connected to body image, which involves thoughts, feelings and behaviours related to one's appearance and functioning. Body image disturbance resulting from cancer and its treatment includes important sexual aspects of altered appearance, sensory changes and functional impairment [7].

BRC and its treatments affect sexual health through several mechanisms. Surgery is the first-line treatment for BRC, in which breast tissues are removed. For most women, breasts and nipples are integral to their feminine identity, and they also play important roles in sexual excitement [3]. Patients report disrupted body image, and various sexual problems after surgery including loss of breast sensation, less sexual desire and arousal, and painful sensations over the breast, chest and shoulders which interfere with sexual activity [8,9]. Adjuvant therapies – chemotherapy, radiation therapy and endocrine therapy – also have adverse effects. Chemotherapy alters body image through hair loss and impairs sexual desire and arousal due to cytokine-related fatigue [10,11]. Chemotherapeutic agents also induce oestrogen blockade in premenopausal women leading to vaginal dryness, decreased sexual desire, painful intercourse and difficulty in reaching orgasm [3,8,9,12]. Radiation therapy can cause skin burning and scarring of the breast, which adversely affects body image and sexual activity. Endocrine therapy decreases sexual desire and causes vaginal dryness, which impair sexual function. These changes all adversely affect intimacy and relationship quality [8]. Such problems are found to be more prominent in BRC women below 50 years because of medication-induced menopause and possibly due to more aggressive and toxic treatments [3,13].

Women with BRC under age 50 report more treatment-related side effects and are more vulnerable to long-term sequelae [3,14]. Of the BRC cases in Hong Kong, around 34% are diagnosed under age 50, which is the median age women reach menopause [15,16]. After completion of BRC treatment, only 22% of premenopausal women had a natural menopause while the others encountered medication-induced menopause with multiple sexual problems including lack of sexual desire, vaginal dryness, and difficulty with arousal, enjoyment or orgasm. These problems often persist beyond five years post-treatment [14]. Living with BRC and its aftermath therefore requires considerable sexual adjustment to re-establish and maintain a satisfying sexual relationship.

Partners of cancer patients also report many challenges after diagnosis and treatment. A study recruited 122 partners, including both male and female to explore the impact of cancer and its treatment on their sexuality and sexual relationships [17]. Most partners (78%) of women with BRC reported alterations in sexuality, with complete cessation of sex or a marked reduction in the frequency of sex after treatment. Perceptions of loss of intimacy made them feel sad. However, only 20% of the

participants were afforded the opportunity to discuss sexuality with a healthcare professional [17]. Intimacy is a key component of relationship quality and could buffer psychological distress [18]. Partners experience psychological distress but their sexual health needs are neglected [17,19]. During treatment, partners are critical to helping women adjust to their illness and offer invaluable support [17,18]. Partners assume the role of carers and people with cancer are repositioned as patients, which subsequently influences sexual relationships due to exhaustion from the caring role, and can lead to relationship discord [12,17]. In addition, as resumption of usual sexual function after treatment for BRC can be difficult, or is sometimes impossible, renegotiation of sexual health with involvement of a supportive partner, is needed to facilitate adjustment to illness [12,17,19], help rebuild intimate relationships and thus improve quality of life (QoL) [20].

Couple-based interventions have yielded significant benefits for sexual health outcomes in other fields and warrant investigation in the BRC context [8,18,21]. Couple-based interventions refer to intervention delivered to couples currently married or living together [21]. Evidence from research on the effects of couple-based interventions for sexual problems following BRC has demonstrated positive results on improving sexual function in terms of orgasm and intimacy, sexual and relationship satisfaction, body image, couple communication, dyadic coping, adjustment and QoL of both patients and their partners [8,18,21]. Providing and receiving support from each other during couple-based interventions promotes joint problem-solving and shared coping [18]. Positive partner coping enhances sexual adjustment and achieves a positive couple relationship [21]. Conventionally, couple-based interventions are delivered in face-to-face mode. However, this format is restricted in pandemic situations. With the popularity and increased recognition of electronic usage of healthcare services, online-based or mobile interventions are viable alternatives to face-to-face delivery of sexual health care [13,22]. It is suggested to be more effective if the delivery mode is adjusted to the needs of patients. In view of the importance of establishing a rapport between clinician and patient, a combination of face-to-face and online modes would be appropriate to address sexual health concern in a comfortable atmosphere [13]. No study has investigated the effects of a multimodal couple-based intervention, with combination of face-to-face and online modes, on sexual health outcomes, and no similar study can be found in PubMed, Cochrane Library or trial registries (ClinicalTrials.gov and ISRCTN).

Given that the previous type and quality of sexual activity is often altered after treatment for BRC [12,17,19], flexibility in sexual renegotiation is regarded as a central aspect of couple-based interventions to address sexual health concern in the context of cancer [19]. The Model of Flexible Coping with Sexual Concerns provides the philosophical underpinning of the proposed intervention. It enables responsive intervention delivery, adapted to need, by expanding the conceptualisation of sexual function and activity, adopting positive coping strategies, and shifting the focus on sexual functioning to intimacy, subsequently bringing beneficial effects on sexual adjustment and relationship satisfaction [23]. It is posited that discussion of flexibility in coping with sexual health concern early in the treatment trajectory facilitates sexual adjustment and the renegotiation of intimate relationships during survivorship. The intervention components integrate evidence-based techniques from cognitive behavioural couple therapy and sex therapy for intimacy enhancement [24], combined with psychoeducation, skills training and therapeutic counselling [21], in order to motivate the change of sexual function and activity in cognitive and behavioural aspects [23]. With flexible coping with sexual concern, individuals can think about sexual activity differently and engage in attainable sexual activities to maintain and foster intimacy [23]. The causal model is illustrated in Appendix A.

In summary, women treated for BRC, particularly those diagnosed and treated premenopausally, and their partners, report many persistent sexual problems and experience relationship challenges post-treatment. Sexual health issues are complex and interrelated and reciprocally influence intimacy and relationship quality [8]. Sexual health and intimacy have significant implications for QoL that determines overall wellbeing [13,19]. Therefore, WHO emphasises that sexual health warrants attention in cancer

care so that QoL can be improved [8]. However, sexual health is a neglected area in clinical practice, for both patients and partners. It is timely to develop an empirically-based and theory-driven flexible couple-coping intervention to address sexual health concern among premenopausal BRC patients and their partners in Hong Kong.

The study is led by an established team of researchers and nurse clinicians who specialise in cancer care and are committed to the wellbeing of women after cancer treatment. The principal investigator (PI) and co-investigators (Co-Is) have worked and published together in cancer research for many years to explore sexual health issues and to develop interventions to improve psychosexual wellbeing in women treated for gynaecological cancer [25-30], with notable success in recruitment, implementation and translatable outcomes. Previous collaborations amongst PI and Co-Is underpin all aspects necessary for this study. Collectively, the team has access to oncology clinic of many public hospitals in Hong Kong. As to methodology, the PI has obtained collaborative support from the Co-Is and a statistician with expertise in quantitative and qualitative data analysis as a Co-I in this study. As to professional qualifications, the PI has postgraduate qualifications in sexuality counselling and psychotherapy.

ii. Research questions

The number of premenopausal women with BRC is growing. These women and their partners experience persistent treatment-related sexual health concerns and unmet sexual health needs. This study aims to develop and implement a novel multimodal couple-coping intervention (MCI) for this cohort in Hong Kong. The research questions are, compared to controls:

1. What are the effects of the MCI on the primary outcome of sexual adjustment in premenopausal BRC women after completion of the intervention, three-month and six-month post-intervention?
2. What are the effects of the MCI on the secondary outcomes of relationship quality and QoL in premenopausal BRC women and their partners after completion of the intervention, three-month and six-month post-intervention?
3. What are participants' experiences of, and compliance with the intervention?

iii. Research methods

A phased-approach will develop, implement and evaluate the MCI. As the newly developed MCI consists of multiple components which interact with each other to achieve the desirable outcomes, it is regarded as a bundled (i.e., complex or multicomponent) intervention [31]. In order to develop an evidence-based complex intervention with an appropriate theory and evaluated rigorously, the Medical Research Council framework is used to guide the process.

Developing a complex intervention

In this phase, the existing evidence on similar interventions, theoretical framework underpinning the interventions, and the changes of outcome to be expected should be identified [31]. This phase has been conducted and the development of the MCI is based on scientific evidence, theoretical framework and the success of our previous GRF-supported project in women with gynaecological cancer.

According to the Model of Flexible Coping with Sexual Concern [23], a more flexible perception of sexuality for individuals who may not be able to resume or engage in their previous level and type of sexual activity is beneficial. In the context of BRC, enhancing the level of flexibility among women treated for BRC and their partners has potential to promote their sexual adjustment, improve relationship quality and QoL. To increase flexibility, the intervention will adopt three strategies: expanding conceptualisation of sexual function and activity, adopting positive coping strategies, and focusing on intimacy [23]. The components of the MCI include psychoeducation, skills training and therapeutic counselling with the integration of techniques from cognitive behavioural couple therapy and sex therapy [21,24]. The contents and design of the intervention are also guided by the findings of previous studies

[18,21,23,24], and the completed GRF project led by the PI in which factors that might enhance the provision of sexuality care and the information needs of sexual health and preferences for sexuality care of female gynaecological cancer patients were identified [25]. The information needs include possibility and suitable timing of sex life resumption, its potential impact on physical health, adaptation to changes in sex life, and potential impact of disease and treatment on physical, psychological and sexual functioning. Regarding the format, the participants indicated the preference for face-to-face counselling with partner participation during post-operation and rehabilitation, and information delivered via internet with reading materials [25].

The proposed MCI will be an eight-week five-session programme, each session delivered every two weeks (at Weeks 0, 2, 4, 6, and 8), and covering topics on the impact of BRC on sex and intimacy, communication and problem-solving skills, dyadic positive coping, cognitive restructuring of sex and intimacy, intimacy-building activities, and maintaining intimacy [18,21,23-25]. The first and last sessions will be conducted in face-to-face dyad format, the second session will be virtual dyad counselling, the third one will be online reading with chat-based discussion forum and homework exercise, and the fourth will be reminder telephone call for reviewing what has been achieved and scheduling the last session. The counselling sessions will last for 60-90 minutes and delivered by a full-time research nurse who will receive one-week training from the PI to provide the intervention, while the online discussion will be moderated by the research nurse to monitor the progress of sensate focus practice which is a common technique used in sex therapy to improve intimacy and communication between partners around sex. The telephone call will last for around 30 minutes. Components and contents of the MCI are listed in Appendices A and B.

Phase I: Refinement of intervention

Design: In this phase a qualitative approach will systematically explore the acceptability and relevance of the MCI from the perspective of Hong Kong premenopausal women treated for BRC and their partners, a panel of experts including academics specialising in oncology research, and healthcare professionals working in oncology units.

Sample size: As recommended by Creswell [32], an expert in mixed-method and qualitative research, 20 to 30 participants form an adequate sample to explore the breadth of a topic in its entirety. We will therefore recruit 10 purposely sampled participants with BRC and their partners (n=20), and 10 experts to form an expert panel for this phase of the study.

Setting and subjects: We will recruit four purposive samples: women treated for BRC, their partners, academics specialising in oncology research, and healthcare professionals working in oncology units. A cash allowance of HK$200 will be given to each dyad to cover travel expenses on completion of this phase of the study.

i. *Women treated for BRC:* Participants will be recruited from the oncology clinic during their regular follow-ups. The inclusion criteria for recruitment are: women with a diagnosis of BRC; all active treatment completed but endocrine and/ or maintenance therapy allowed; with a regular sexual partner, either heterosexual or homosexual; over 18 years; in a premenopausal state when diagnosed with BRC; has a smartphone with internet connection; able to understand spoken Cantonese and to read Chinese; consenting to participate. Those with a known pre-existing psychiatric illness will be excluded.
ii. *Partners of BRC women:* Partners of the participating patients will be recruited.
iii. *Academics:* Academics who specialise in oncology research will be recruited from the institute of the PI. There are six academics working on this research theme and all of them will be invited to join the interviews.
iv. *Healthcare professionals:* Registered nurses and physicians working in the oncology unit of two participating hospitals, with at least two-year clinical experience in the specialty, will be invited to

join the interviews. Their views and opinions on the MCI would contribute to the accessibility and sustainability of implementation of the intervention in important ways.

Procedure: Eligible women will be approached and invited to participate in the study. The first session of the MCI will be delivered after recruitment if the partner is present, or on another schedule. The other four sessions will then be conducted every two weeks. On the completion of the last face-to-face counselling session, a semi-structured interview will be conducted to explore the acceptability and comments on the MCI. An interview guide will be adopted with examples of questions: 'What's your general impression of the programme?' 'How did the intervention help you cope with changes in sex and intimacy after treatment for BRC?' and 'Describe for me how you set goals for sexual adjustment as a result of this programme.' Participants will be encouraged to provide comments on the intervention. Based on previous experience with this cohort, each interview should last around one hour.

For the participating academics and healthcare professionals, the intervention components, content and design will be illustrated to them during individual interviews at the hospitals or in other venues convenient to the participants. They will be invited to provide comments and suggestions on the intervention, as well as its implementation and sustainability.

Data analysis: All interviews will be audio-taped and transcribed verbatim by the research nurse conducting the interviews and verified by the PI against the taped record. The PI, and a Co-I who has rich experience in conducting qualitative research, will carry out data analysis independently by means of content analysis. The PI will discuss any discrepancy in coding with the Co-I and other investigators to achieve resolution. The findings will be used to refine the intervention for next phase of the study.

Phase II: Evaluating a complex intervention

Design: In this phase an assessor-blind, parallel randomised controlled trial (RCT) will be conducted to evaluate the effects of the MCI on sexual adjustment as the primary outcome among premenopausal women treated for BRC, relationship quality and QoL as secondary outcomes among the women and their partners. There are three research hypotheses: participants in the intervention will have better 1) sexual adjustment, 2) relationship quality, and 3) QoL, after the completion of the intervention than those in the attention control group.

Sample size: The sample size is determined to give the study adequate power to evaluate the effects of the MCI on the outcomes of sexual adjustment, relationship quality and QoL. To our knowledge, no study has investigated the effects of a multimodal couple-based intervention on sexual health outcomes. Without an empirically-derived effect size to guide our sample size planning, we instead define a clinically relevant change as a 0.5 standard deviation (SD) improvement in the above outcomes on *a priori* basis. Such an effect corresponds to a medium effect size [33] and is empirically regarded as a minimally important difference [34]. By using the power analysis software PASS 16 (NCSS, LLC. Kaysville, Utah, USA), it is estimated that a sample size of 64 dyads per group is adequate to achieve 80% power at two-sided 5% level of significance to detect an effect size of 0.5 SD in an outcome at a post-intervention time point. Further allowing for an overall attrition rate up to 20% [21], a total of 160 eligible dyads with 80 per group will be recruited into the RCT.

Settings and subjects: The inclusion criteria for women treated for BRC and the recruitment process described in Phase II will apply to this phase, but subjects will be women completed active treatment for BRC in recent six months but endocrine and/ or maintenance therapy allowed, and with no evidence of metastatic disease, so that the intervention will be delivered to them during early survivorship, with the intent to prepare the women and their partners for life after cancer. Partners of the eligible subjects will be recruited as in dyad. HK$150 cash coupon will be given to each participant for time compensation on completion of all reassessments.

Procedure: Eligible women and their partners will be approached in the oncology clinic and have the aims of the study explained to them. Information sheets about the study and consent forms will be

provided. After written consent is obtained, the participating dyad's demographic data will be collected and the instruments administered during baseline face-to-face interviews. The participants will then be randomly assigned to either the intervention or attention control group. Subject allocation will be done using stratified block randomisation with a block size of 10. A random sequence of grouping identifiers (I or C), based on computer-generated random numbers, will be prepared in advance by an independent statistician. The grouping sequence lists will be password-protected and stored in a computer, and only be accessible to staff independent of the study or responsible for group allocation. The group allocation of each participant will be assigned according to the sequence of enrolment and the corresponding group identifier in the previously prepared random sequence list. The trained research nurse will deliver the MCI to the participants and their partners. In order to ensure the fidelity of the intervention, the PI will monitor the intervention delivered to the first 10 dyads. Reflective notes and a record of the participants' attendance will be kept as a means of process evaluation. To ensure blinding of the outcome assessor, a part-time research assistant who will not be informed of the group status of the participants will be responsible for reassessment. Reassessment will be conducted at the same time point for both groups, i.e. on the completion of the intervention, three months and six months post-intervention via telephone call. Participating dyads in the intervention group will be invited to be interviewed about their experiences and feelings towards the intervention, and will be audio-taped. Another research nurse will conduct qualitative interviews with the participating dyads to prevent an experimenter effect.

Data collection: Participation and completion rates of the intervention by the participating dyads in the intervention group will be monitored. A demographic and clinical data sheet will be used to record the socio-demographic and clinical characteristics of the participants. Effects of the intervention on the outcome variables will be measured at baseline (T0), on completion of the intervention (T1), three months (T2) and six months (T3) post-intervention by using the following measures:

1. *Chinese version of the sexual adjustment scale of the Sexual Adjustment and Body Image Scale (SABIS)*

The Chinese version of the sexual adjustment scale of SABIS will assess the effect of the MCI on sexual adjustment among the women treated for BRC. The SABIS consists of two scales: a six-item body image scale and an eight-item sexual adjustment scale. Using a five-point Likert scale with scores ranging from 1 to 5, higher scores indicate better outcomes. The scale has been used previously in BRC with good reliability and validity [9]. While no Chinese version is available, we have developed one using the Brislin model of translation and guidelines for cross-cultural adaptation of scales. The translated version of SABIS will be assessed for semantic equivalence and content validity by a panel of six healthcare professionals, with a content validity index of 0.8 or above set as the satisfactory criteria, and revised if necessary, before administration to participants.

2. *Chinese version of ENRICH Marital Satisfaction Scale (EMS)*

The Chinese version of EMS will be used to assess the quality of partner relationships of the participating dyads individually. It consists of 10 items, extracted from the original full-length 125-item ENRICH Marital Inventory, which are recognised as the most important dimensions of relationships by the original author. A five-point Likert scale with scores ranging from 1 to 5 is used, with higher scores indicating better relationships. The scale has established discriminant validity and demonstrated good reliability, with an internal consistency of 0.76 [35].

3. *Chinese version of the MOS 36-item Short Form (SF-36) Health Survey*

QoL of the participating dyads will be measured by the Hong Kong Chinese version of the SF-36. The 36 items are clustered into eight domains: physical functioning (PF), role physical (RP), bodily pain (BP), general health (GH), vitality (VT), social functioning (SF), role emotional (RE), and mental health (MH). All items in each domain are summed and transformed into scales from 0 to 100. Furthermore, the PF, RP, BP, and GH domains are aggregated into the physical health component summary (PCS) score, and the VT, SF, RE, and MH domains are aggregated into the mental health component summary (MCS)

score. Higher scores indicate better QoL. The Hong Kong Chinese version of the SF-36 has demonstrated good construct validity, internal consistency and test-retest reliability among Chinese patients in primary care [36].

4. <u>Demographic and clinical data sheet</u>

A data collection sheet will record the socio-demographic and clinical characteristics of the BRC women: age, education level, monthly household income, marital status, duration of marriage/current relationships, number of children, religious beliefs, stage of cancer, time since diagnosis, treatment modality and length of treatment. Socio-demographic data of the partners will also be collected: age, education level, medical and drug history.

Qualitative data will be collected from the intervention group through semi-structured interviews up to the point of data saturation, estimated to be 20 participating dyads. Their experiences and perceptions of the MCI, and whether the programme improved their sexual adjustment will be explored. The interviews will be scheduled after the completion of the programme, and audiotaped with consent.

Data analysis: Data will be summarised and presented using appropriate descriptive statistics. The homogeneity of participants' characteristics between the two study arms will be assessed using independent t, Mann-Whitney, chi-square or Fisher's exact tests, as appropriate. Intention-to-treat principle will be adopted in the outcome analysis by using a generalised estimating equations (GEE) model to compare the differential changes in each outcome across the four time points between the two arms. All statistical analyses will be performed using SAS release 9.4 (SAS Institute, Cary, NC.). All statistical tests are two-sided with level of significance set at 0.05. Audiotapes of the interviews will be examined by means of content analysis. First, the recorded tapes will be transcribed verbatim. All transcripts will be compared with the recording to check for any discrepancies, and revised as necessary. The transcripts will then be content analysed to identify persistent words and code themes within the data. Finally, significant categories and themes of the data will be formulated and confirmed by the PI and Co-I to improve its accuracy, and then translated into English [37].

Ethical issues

The issue of confidentiality is the major ethical issue, and will be solved by recording the data in a manner that does not allow the participants to be identified (ie. using a non-recognizable code for each patient). Ethical approval will be sought from the Joint Chinese University of Hong Kong and New Territories East Cluster Clinical Research Ethics Committee and the Hong Kong East Cluster Research Ethics Committee. The study is in compliance with the Helsinki Declaration and ICH-GCP. Permission to use the indicated instruments will be obtained from the original authors. The study's purpose, nature and procedures will be explained to the eligible participants. Agreement and consent will be obtained from the participants. All participation will be on a voluntary basis and the participants will have the right to withdraw at any time.

Data handling and record keeping

To protect patient privacy, all research data would be handled in line with HA/Hospital's policy in handling/storage/destruction of patients' medical records. All data will be anonymised and used for research purposes only. The data will be kept confidential in a locked cabinet where the department or ward keeps patients' confidential information and on a password-protected computer for no more than 10 years after the study and will then be destroyed. All hard copies of survey will be kept for 5 years and will be destroyed thereafter. The principal investigator will be responsible for safekeeping of the personal data during and after the study. The whole investigator team and the employed research staff will have access to the data or study record during and after the study.

Financing and Insurance

The study has been granted by a local research grant, General Research Fund to support the staff cost and the expenses on the project. The study will not consume any resources from the Hospital Authority and the subjects will not be charged for the study. All participants except the academics and healthcare professionals will receive cash incentives HK$200 after the completion of Phase I study. In Phase II study, HK$150 cash coupon will be given to each participant for time compensation on completion of all reassessments.

The issue of insurance is not applicable in this study.

Publication Policy
The study will be registered in a public domain trial registry, ISRCTN registry, BMC. The Lead PI will be responsible to do that.

References

1. Centre for Health Protection, Department of Health, The Government of the Hong Kong Special Administrative Region. Breast cancer; 2021 May 20 [cited 2021 Sep 7]. Available from https://www.chp.gov.hk/en/healthtopics/content/25/53.html
2. Runowicz CD, Leach CR, Henry NL, Henry KS, Mackey HT, Cowens-Alvarado RL, Cannady RS, Pratt-Chapman ML, Edge SB, Jacobs LA, Hurria A, Marks LB, LaMonte SJ, Warner E, Lyman GH, Ganz PA. American cancer society/American society of clinical oncology breast cancer survivorship care guideline. Cancer J Clin. 2016 Jan-Feb;66(1):43-73.
3. Jeng CJ, Hou MF, Liu HY, Wang LR, Chen JJ. Construction of an integrated sexual function questionnaire for women with breast cancer. Taiwan J Obstet Gynecol. 2020 Jul;59(4):534-40.
4. World Health Organisation. Sexual health throughout life: Definition; 2021 [cited 2021 Sep 7]. Available from https://www.euro.who.int/en/health-topics/Life-stages/sexual-and-reproductive-health/news/news/2011/06/sexual-health-throughout-life/definition
5. Woods NF. Toward a holistic perspective of human sexuality: alterations in sexual health and nursing diagnoses. Holist Nurs Pract. 1987 Aug;1(4):1-11.
6. Chen CH, Lin YC, Chiu LH, Chu YH, Ruan FF, Liu WM, Wang PH. Female sexual dysfunction: definition, classification, and debates. Taiwan J Obstet Gynecol. 2013 Mar;52(1):3-7.
7. Fingeret MC, Teo I, Epner DE. Managing body image difficulties of adult cancer patients: lessons from available research. Cancer. 2014 Mar;120(5):633-41.
8. Carroll AJ, Baron SR, Carroll RA. Couple-based treatment for sexual problems following breast cancer: a review and synthesis of the literature. Support Care Cancer. 2016 Aug;24(8):3651-9.
9. Dalton EJ, Rasmussen VN, Classen CC, Grumann M, Palesh OG, Zarcone J, Kraemer HC, Kirshner JJ, Colman LK, Morrow GR, Spiegel D. Sexual Adjustment and Body Image Scale (SABIS): a new measure for breast cancer patients. Breast J. 2009 May-Jun;15(3):287-90.
10. Bower JE, Ganz PA, Aziz N, Fahey JL. Fatigue and proinflammatory cytokine activity in breast cancer survivors. Psychosom Med. 2002 Jul-Aug;64(4):604-11.
11. Oflazoğlu U, Varol U, Alacacıoğlu A, Aşık N, Salman T, Taşkaynatan H, Küçükzeybek Y, Yıldız Y, Tarhan MO. The effect of adjuvant chemotherapy on sexual satisfaction and quality of life in breast cancer patients and their partners Izmir Oncology Group (IZOG) study. Acta Oncologica Turcica. 2018 Jan;51(3):357-62.
12. Mendoza N, Molero F, Criado F, Cornellana MJ, González E. Sexual health after breast cancer: recommendations from the Spanish Menopause Society. Maturitas. 2017 Nov;105:126-31.

13. Kang HS, Kim HK, Park SM, Kim JH. Online-based interventions for sexual health among individuals with cancer: a systematic review. BMC Health Serv Res. 2018 Mar;18(1):167.
14. Bloom JR, Stewart SL, Chang S, Banks PJ. Then and now: quality of life of young breast cancer survivors. Psychooncology. 2004 Mar;13(3):147-60.
15. Hong Kong Cancer Registry, Hospital Authority. Carcinoma in-situ (Ca in-situ) of female breast; 2020 [cited 2021 Sep 7]. Available from https://www3.ha.org.hk/cancereg/pdf/factsheet/2018/breast_2018.pdf
16. Family Health Service, Department of Health, The Government of the Hong Kong Special Administrative Region. When does menopause usually take place; 2021 Jan 4 [cited 2021 Sep 7]. Available from https://www.fhs.gov.hk/english/health_info/faq/women_health/WH2_5_5.html
17. Hawkins Y, Ussher J, Gilbert E, Perz J, Sandoval M, Sundquist K. Changes in sexuality and intimacy after the diagnosis and treatment of cancer: the experience of partners in a sexual relationship with a person with cancer. Cancer Nurs. 2009 Jul-Aug;32(4):271-80.
18. Zimmermann T. Intimate relationships affected by breast cancer: interventions for couples. Breast Care (Basel). 2015 Apr;10(2):102-8.
19. Gilbert E, Ussher JM, Perz J. Renegotiating sexuality and intimacy in the context of cancer: the experiences of carers. Arch Sex Behav. 2010 Aug;39(4):998-1009.
20. Moreira H, Canavarro MC. Psychosocial adjustment and marital intimacy among partners of patients with breast cancer: a comparison study with partners of healthy women. J Psychosoc Oncol. 2013 May;31(3):282-304.
21. Li Q, Loke AY. A systematic review of spousal couple-based intervention studies for couples coping with cancer: direction for the development of interventions. Psychooncology. 2014 Jul;23(7):731-9.
22. Karim H, Choobineh H, Kheradbin N, Ravandi MH, Naserpor A, Safdari R. Mobile health applications for improving the sexual health outcomes among adults with chronic diseases: a systematic review. Digit Health. 2020 Jan-Dec;6:2055207620906956.
23. Reese JB, Keefe FJ, Somers TJ, Abernethy AP. Coping with sexual concerns after cancer: the use of flexible coping. Support Care Cancer. 2010 Jul;18(7):785-800.
24. Reese JB, Zimmaro LA, Lepore SJ, Sorice KA, Handorf E, Daly MB, Schover LR, Kashy D, Westbrook K, Porter LS. Evaluating a couple-based intervention addressing sexual concerns for breast cancer survivors: study protocol for a randomized controlled trial. Trials. 2020 Feb;21(1):173.
25. Chow KM, Chan CWH, Choi KC, White ID, Siu KY, Sin WH. A practice model of sexuality nursing care: a concept mapping approach. Support Care Cancer. 2021 Mar;29(3):1663-73.
26. Chow KM, Chan CWH, Law BMH. Perceptions of Chinese patients treated for gynaecological cancer about sexual health and sexual information provided by healthcare professionals: a qualitative study. Cancers (Basel). 2021 Apr;13(7):1654.
27. Chow KM, Chan CWH, Choi KC, Siu KY, Fung HKS, Sum WM. A theory-driven psycho-educational intervention programme for gynaecological cancer patients during treatment trajectory: a randomised controlled trial. Psychooncology. 2020 Feb;29(2):437-43.
28. Chow KM, So WKW, Choi KC, Chan CWH. Sexual function, psychosocial adjustment to illness, and quality of life among Chinese gynaecological cancer survivors. Psychooncology. 2018 Apr;27(4):1257-63.

29. Chow KM, Chan CW, Chan JC, Choi KK, Siu KY. A feasibility study of a psychoeducational intervention program for gynecological cancer patients. Eur J Oncol Nurs. 2014 Aug;18(4):385-92.
30. Chow KM, So WKW, Chan CWH. Assessing sexual function amongst Hong Kong Chinese patients with gynecological cancer: translation and validation of the Sexual Function-Vaginal Changes Questionnaire (SVQ). Clin Oncol Cancer Res. 2010 Aug;7(4):230-3.
31. Craig P, Dieppe P, Macintyre S, Michie S, Nazareth I, Petticrew M. Developing and evaluating complex interventions: the new Medical Research Council guidance. Int J Nurs Stud. 2013 May;50(5):587-92.
32. Creswell JW. Qualitative inquiry and research design: choosing among five traditions. Thousand Oaks, CA: Sage Publications; 1998.
33. Cohen J. A power primer. Psychol Bull. 1992 Jul;112(1):155-9.
34. Norman GR, Sloan JA, Wyrwich KW. Interpretation of changes in health-related quality of life: the remarkable universality of half a standard deviation. Med Care. 2003 May;41(5):582-92.
35. Shen ACT. The applicability of Western marital satisfaction measures for couples in Taiwan based on ENRICH. Psychol Test. 2001 Jan;48(2):131-51.
36. Lam CLK. Reliability and construct validity of the Chinese (Hong Kong) SF-36 patients in primary care. Hong Kong Pract. 2003 Oct;25(10):468-75.
37. Twinn S. An exploratory study examining the influence of translation on the validity and reliability of qualitative data in nursing research. J Adv Nurs. 1997 Aug;26(2):418-23.
38. World Health Organisation. Breast cancer now most common form of cancer: WHO taking action; 2021 [cited 2021 Sep 7]. Available from: https://www.who.int/news/item/03-02-2021-breast-cancer-now-most-common-form-of-cancer-who-taking-action
39. Hong Kong Cancer Registry, Hospital Authority. Overview of Hong Kong cancer statistics of 2018; 2020 [cited 2021 Sep 7]. Available from: https://www3.ha.org.hk/cancereg/pdf/overview/Overview%20of%20HK%20Cancer%20Stat%202018.pdf
40. Sun F. Covid-19 toll on marriage: divorce inquiries on the rise as stay-home measures push Hong Kong couples off the edge [Internet]. 2020 May 10 [cited 2021 Sep 7]; South China Morning Post. Available from: https://www.scmp.com/news/hong-kong/health-environment/article/3083681/covid-19-toll-marriage-divorce-inquiries-rise
41. Zee B, Huang C, Mak S, Wong J, Chan E, Yeo W. Factors related to sexual health in Chinese women with breast cancer in Hong Kong. Asia Pac J Clin Oncol. 2008;4(4):218-26.
42. The Government of the Hong Kong Special Administrative Region. Hong Kong Cancer Strategy; 2019 [cited 2021 Sep 7]. Available from: https://www.fhb.gov.hk/download/press_and_publications/otherinfo/190700_hkcs/e_hkcs_fully.pdf
43. Hong Kong Breast Cancer Foundation. Hong Kong Breast Cancer Registry Report No.12; 2020 [cited 2021 Sep 7]. Available from https://www.hkbcf.org/en/our_research/main/519/
44. Kalaitzi C, Papadopoulos VP, Michas K, Vlasis K, Skandalakis P, Filippou D. Combined brief psychosexual intervention after mastectomy: effects on sexuality, body image, and psychological well-being. J Surg Oncol. 2007 Sep;96(3):235-40.
